# A QR code-based user-friendly visual cryptography scheme

**DOI:** 10.1038/s41598-022-11871-9

**Published:** 2022-05-10

**Authors:** Lijing Ren, Denghui Zhang

**Affiliations:** 1 Cyberspace Institute of Advanced Technology, Guangzhou University, Guangzhou, 510006 People’s Republic of China; 2 School of Traffic and Transportation, Shijiazhuang Tiedao University, Shijiazhuang, 050043 People’s Republic of China

**Keywords:** Biomedical engineering, Mechanical engineering

## Abstract

Benefiting from the development of the Internet and smart devices, it is now convenient to transmit images anywhere and anytime, which poses a new challenge for image security. The Visual Cryptography Scheme (VCS) is a secret sharing method for protecting an image without a key, the merit of VCS is the human visual system (HVS) can restore the secret image by simply superimposing qualified shares, without any computation. To eliminate noise-like shares in traditional VCS, this paper presents a novel QR code-based expansion-free and meaningful visual cryptography scheme (QEVCS), which generates visually appealing QR codes for transmitting meaningful shares. When distributing on public networks, this scheme does not attract the attention of potential attackers. By limiting the gray-level of a halftoned image, QEVCS both keep the computation-free of visual cryptography and the size of recovery image same as the secret images. The experimental results show the effectiveness of QEVCS when preserving the privacy of images.

## Introduction

In recent years, the rapid development of smart devices and 5G technologies have had a great impact on all walks of life, now people are enjoying conveniences brought by Internet services. As an important information carrier, digital images are widely used in fields including pattern recognition, virtual reality, and medical imaging^1^. The universality poses new challenges for personal privacy. With information leakage accidents emerging, it is urgent to protect important information in digital images^2,3^. Although the traditional cryptography, watermarking, and steganography techniques can protect sensitive information by encryption^4,5^, the encryption and decryption processes are computationally intensive and require a lot of effort to the keys in these schemes.

Secret sharing is a scheme to split a secret into multiple shares, and each share is managed by different participants. Only qualified participants can collaborate to recover the secret message, while a single participant reveals nothing about the secret message. VCS is one of the secret-sharing methods for image security, which was first proposed by Naor and Shamir^6^. Since then, it has received widespread attention from researchers. The merit of VC lies in that HVS can restore the secret image by simply superimposing qualified shares, without any digital devices. VCS solves the problems of key management in traditional cryptography and provides a simple and effective method for distributed storage of images. However, VCS has suffered two drawbacks: (1) *pixel-expansion*: due to the use of subpixels with multiple pixels to encrypt a single secret pixel, the size of shared images is larger than the original image; (2) *meaningless*, VC protects a secret image by sharing it into noise-like shares. The shares prevent information leakage. However, it is difficult to distinguish each other and brings a burden for the management of noise-like shares.

Quick Response (QR) code is a kind of popular two-dimensional barcode^7^, which is a machine-readable optical label with the advantages of speed reading, error correction ability, rich data formats^8^. Benefitting from the development of the mobile Internet, QR code is widely used to transmit complex digital information in the physical world, such as payment information, contact cards, and advertisements.

The appearance of the QR code is similar to the share of VCS, which both are black-and-white images (binary image). QR codes provide a suitable carrier for the transmission of shares. Researchers have put forward many contributions to aggregate the advantages of VCS and QR codes. Pan et al.^9^ use four or more color QR codes to generate meaningful shares based on the color XOR scheme. Although the proposed scheme can fully restore a secret image, it still needs a meaningless share to meet the XOR operation constraint. Wan et al.^10^ presented a scheme to alter the bits corresponding in the range of the error correction mechanism. HVS can reveal the secret image by stacking. When the computation is available, it can reveal a better visual quality image based on the XOR operation. To meet the error correction conditions, the larger the secret image, the more share images are generated. Tan et al.^11^ proposed an XOR-based VCS applying to grayscale QR codes. The scheme substitutes a bit of a share for the second significant bit of the QR code cover image, which can resist common image attacks. Cheng et al.^12^ designed an innovative two-level QR code that takes advantage of the concentric feature in the QR code and replaces a module with a cell with $3\times 3$ sub-modules. The concentric sub-module contains the public message while the remaining store secret messages. The scheme can recover secret messages with XOR and threshold operations. Cheng et al.^13^ presented a novel scheme for ($n,n) \left(n\ge 3\right)$ threshold to improve the security of QR codes with XOR-based VCS. The proposed scheme further extended the access structure from $(n,n)$ to $(k,n)$ by the error correction mechanism of QR codes.

The aforementioned schemes cannot restore the secret image by simply superimposing images like the original VCS due to the XOR operation. However, it is still expected to reveal images directly through HVS in many scenarios including medical images and paper maps^14^. Our scheme can be applied to these scenes without cryptographic computation.

In this paper, we propose a QR code-based VCS to address the pixel-expansion and meaningless issues in VCS. First, we design a block-by-block extended VCS (EVCS, also called user-friendly VCS) to archive meaningful shares on the premise of keeping the size-invariant of secret images, thereby avoiding suspicion in potential attackers. With the OR operation, HVS can directly reveal the secret image by simply overlapping received shares. Then, we use the rich data capacity of QR codes to transmit shares. The encoded QR codes do not destroy the error correction codewords and can be scanned and decoded by a normal QR code reader. QECVS realizes the secure transmission of secret images without destroying the advantages of QR codes and VCS.

## QEVCS: a QR code-based expansion-free extended visual cryptography scheme

**Figure 1:**
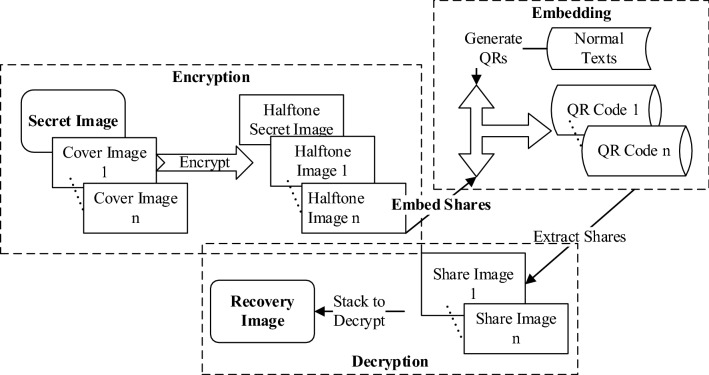
The encryption and decryption architecture of the proposed QEVCS.

The ideas behind QEVCS are (1) keeping the size-invariant when archiving meaningful shares of an image; (2) transmitting shares using QR codes. As shown in Fig. 1, QEVCS divides the encryption process of images into two parts. Firstly, we propose a gray-level limited EVCS, which splits a secret image into two equal-sized and meaningful cover images, and then embed cover images into coressponding QR codes images by using the gray decoding and concentric decoding characteristics of QR codes, so as to realize the QR codes transmission of a secret image. We will first introduce the proposed expansion-free EVCS.

In the original EVCS, to encrypt a single pixel, it needs a sub-pixel composed of multiple pixels for secret sharing. A common method is block-wise encryption^14,15^ for eliminating pixel expansion. The basic idea of block encryption is to encrypt by sharing blocks whose size is equal to that of the secret block. To eliminate the same pixel expansion problem in the traditional EVCS model, we use a block-based halftoning operation instead of pixel-by-pixel encryption to maintain sizes in the secret block and share blocks.

The proposed QEVCS has to ensure that the generated pixel blocks meet the requirement of EVCS encryption during halftoning. Grayscale images have 256 levels, while images generated by VCS only have two grayscale levels of black and white. Therefore, halftone is indispensable to transmit images using EVCS. Algorithm 1 shows the flow of the proposed limited gray-level halftoning algorithm. The whole encoding flow of QEVCS is described in detail in Algorithm 2.

The size of the block ${s}_{b}$ is the same as the pixel expansion value in the ($k,n$)-EVCS. When binarizing a grayscale image, the gray-level ${\mathrm{b}}_{\mathrm{Bs}}$ of the secret block and the gray-levels ${\mathrm{b}}_{\mathrm{B}1}, {\mathrm{b}}_{\mathrm{B}2}$ of cover blocks have to satisfy the following relationship:

1 $${\mathrm{b}}_{\mathrm{Bs}}\in [\mathrm{max}(0,{\mathrm{b}}_{\mathrm{B}1}+{\mathrm{b}}_{\mathrm{B}2}-{\mathrm{s}}_{\mathrm{b}} ),\mathrm{min}({\mathrm{b}}_{\mathrm{B}1},{\mathrm{b}}_{\mathrm{B}2} )]$$

The constraint enables it to reuse the existing EVCS when addressing pixel expansion in original methods. To reduce the loss of image quality, we can adjust the combination of sharing blocks. The algorithm is no longer constrained to produce a binary output with a single threshold but determines the closest allowed visual grayscale to generate an output image of more than two levels.
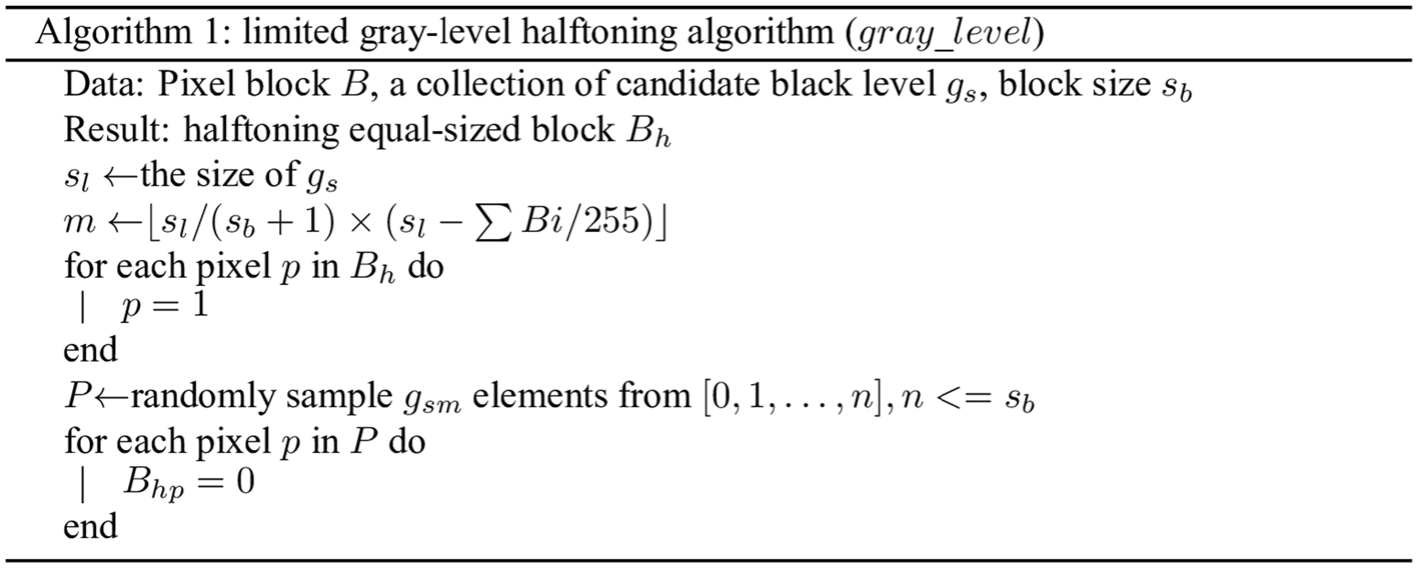

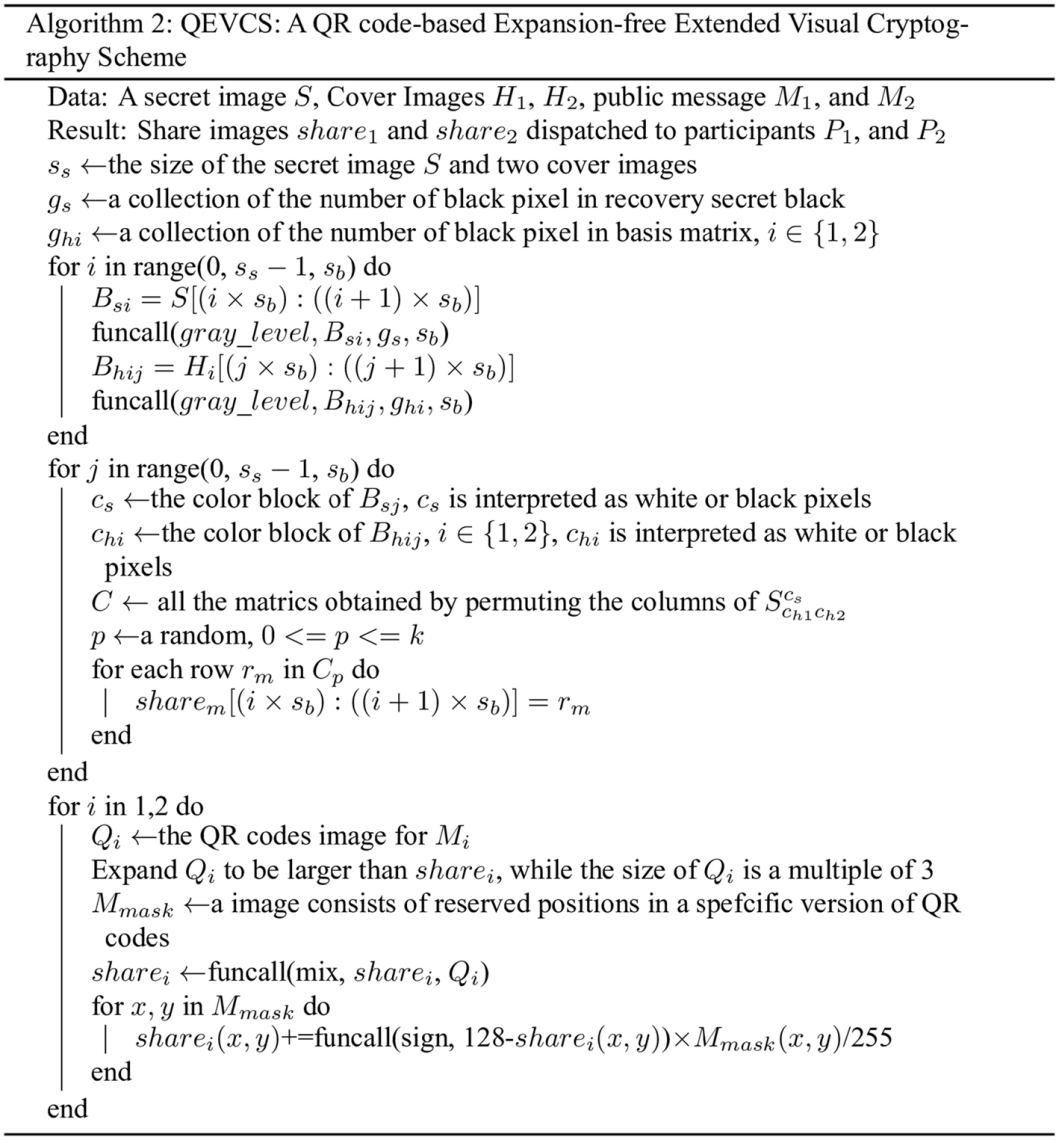


In the encoding process, a grayscale image is first divided into $n$ non-overlapping black-and-white pixel blocks ${\mathrm{B}}_{\mathrm{i}},{\mathrm{B}}_{\mathrm{i}}\cap {\mathrm{B}}_{\mathrm{j}}=\mathrm{\varnothing },\mathrm{ for }1\le \mathrm{i}\ne \mathrm{j}\le \mathrm{n}$. The ${\mathrm{B}}_{\mathrm{i}}$ before halftoning and the block ${\mathrm{B}}_{\mathrm{h}}$ after halftoning are both of the same size. The number of black pixels in ${B}_{i}$ and ${B}_{h}$ has to satisfy the following condition:

2 $${\mathrm{b}}_{\mathrm{Bi}}= \left\lfloor {s_{l} /\left( {s_{b} + 1} \right) \times \left( {s_{l} - \sum {B_{i} } /255} \right)} \right\rfloor$$

where ${\mathrm{s}}_{\mathrm{l}}$ denotes the number of candidate black blocks. For a secret block with the size ${s}_{b}=2\times 2$, the gray level is ranged from ${b}_{Bi}\in [\mathrm{0,1},\mathrm{2,3},4]$ after halftoning. Before converting the original gray image into a black-and-white image, it is necessary to determine the block criterion of the image. In the error diffusion algorithm, all five gray levels may appear, thus producing a halftone image similar to the original image. To ensure the gray-levels of cover blocks after halftoning can meet the requirement for the gray-level of the secret block, we use only a limited number of gray-levels in the chunked halftone set while fixing the ratio of black and white pixels in each block. Taking the (2,2)-EVCS for example, after splitting a secret image into (2,2) blocks, the number of black pixels can only be 3 or 4, that is, ${g}_{s}=[\mathrm{3,4}]$. While the number of black pixels in cover images only be 2 and 3, that is, ${g}_{s}=[\mathrm{2,3}]$. After completing the limited halftone of the secret image and cover images, we can combine the existing EVCS to rearrange the pixels of the halftoned block according to the secret color block.

Note that we do not use error-effusion technology in the *gray_level* method. Limiting gray levels is equivalent to reducing gray values artificially. Using the error-diffusion technology will quickly accumulate the white error of current pixels to adjacent pixels. It will result in the subsequent pixels becoming all-white blocks and generating images with lower quality.

The original purpose of a QR code is to transmit text information. Although the capacity increases with the increasing version, its capacity is still limited relative to the image. It can be seen that the size of the largest QR codes is only $177\times 177$, and the size of an image is much larger than this size. If an image is directly embedded into a QR code, it will destroy the encoding rules of the QR code, which will make it difficult to identify.

3 $$M={\left[\begin{array}{ccc}{p}_{\mathrm{1,1}}& {p}_{\mathrm{1,2}}& {p}_{\mathrm{1,3}}\\ {p}_{\mathrm{2,1}}& {p}_{\mathrm{2,2}}& {p}_{\mathrm{2,3}}\\ {p}_{\mathrm{3,1}}& {p}_{\mathrm{3,2}}& {p}_{\mathrm{3,3}}\end{array}\right]}_{\dots }$$

4 $${p}_{scan}={\tau (M}_{c})$$

The above operation shows the real value read by a reader when denoting a module in QR codes with a block $M$, where $\tau$ is a threshold function, ${M}_{c}$ is the centric pixel in $M$ and ${p}_{scan}$ is the fetched pixel. Based on the centric property of QR codes, we first expand the size of QR codes so that its size is a multiple of the minimum 3 of the secret image. Then we overlay the pixels in the QR code to the position of ${M}_{c}$ in the image pixel-by-pixel, while keeping the other pixels like ${p}_{\mathrm{1,1}}, {p}_{n,n}$ in the cover image unchanged. HVS can still identify the image mixed with QR code and meaningful shares. However, because QR codes will overlay part of the share pixels, we further utilize the gray property of QR codes to embed shared pixels into the least significant bit of QR codes at the corresponding position of cover images:

5 $${P}_{Q}\left(x,y\right)={P}_{Q}\left(x,y\right)+sign\left({128-P}_{Q}(x,y)\right)\times {p}_{e}/255$$

where ${p}_{e}$ is the embedded pixel in the cover images, and ${P}_{Q}(x,y)$ is the corresponding pixel in the QR codes image. If ${p}_{e}$ is a black pixel, ${P}_{Q}(x,y)$ will be unchanged. While if ${p}_{e}$ and ${P}_{Q}\left(x,y\right)$ are both white pixel (255), the gray-level of ${P}_{Q}(x,y)$ will be 254. If ${p}_{e}$ is a white pixel while ${P}_{Q}(x,y)$ is a black pixel, its gray level will be 1. It can be inferred that no matter which the gray-level of a pixel is, the influence on the gray value of the original pixel after the embedding operation does not exceed 1, thus archiving the minimum disturbance to QR codes.

## Experiments and analyses

**Figure 2:**
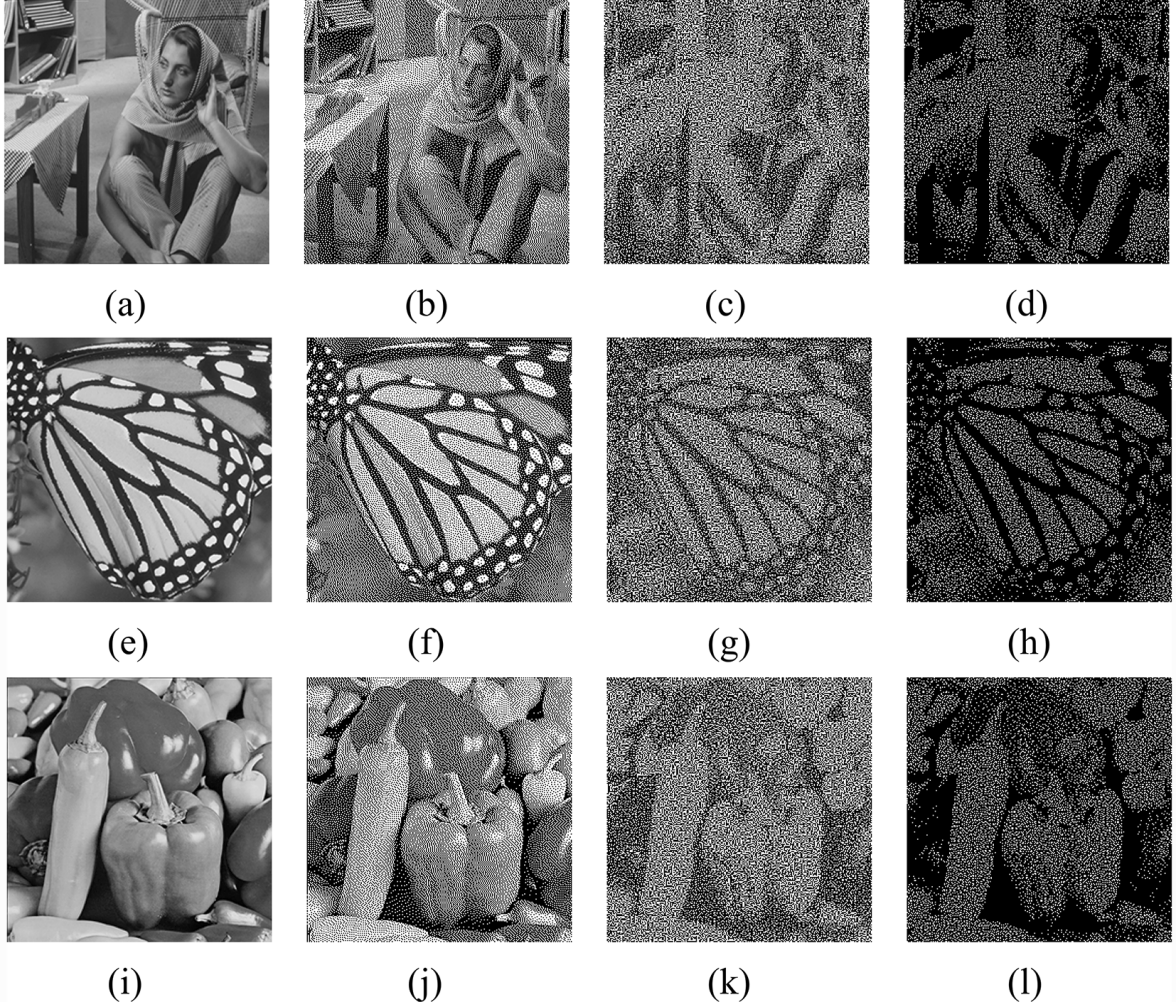
Experimental results of halftone images. Columns are original gray-scale images, halftone images generated by the ER method, halftone images generated by two thresholds, respectively. All the size is $512\times 512$.

In this section, we will evaluate the effectiveness of the proposed QEVCS. Figure 2 shows the experimental result processed with the limited halftone ($gray\_level$). The selected test images are the classic Barbara (Fig. 2a), Butterfly (Fig. 2e), and Peppers (Fig. 2i). The first column is original gray-scale images, the second column (Fig. 2b,f,j) is halftone images generated by the ER method, the third (Fig. 2c,g,k) and fourth (Fig. 2d,h,l) columns are halftone images generated by $gray\_level$ with two thresholds. The difference is that the third column represents black and white pixels with subpixels with 2/4 and 3/4 black pixels respectively, while the fourth column represents black and white pixels with 3/4 and 4/4 black pixels.

The limited halftone scheme reinterprets the color blocks with different proportions of black pixels (or white) into black (or white) pixels, thus matching the underlying EVCS schemes. By comparing images in the third and fourth columns, we can see that the contrast of images varies with the proportion of black and white blocks. There are different levels of image degradation. Because images in the second column adopt ER, the visual effect of the generated image is close to that of the original image. While the proportion of black pixels in the color block is constrained in the fourth column. The removal of the block arrangement with 4, 3, and 2 white pixels leads to a degradation of the quality of the recovered image, it can be seen that the recovered image is darker. However, we can still see the features of the original images.

**Figure 3:**
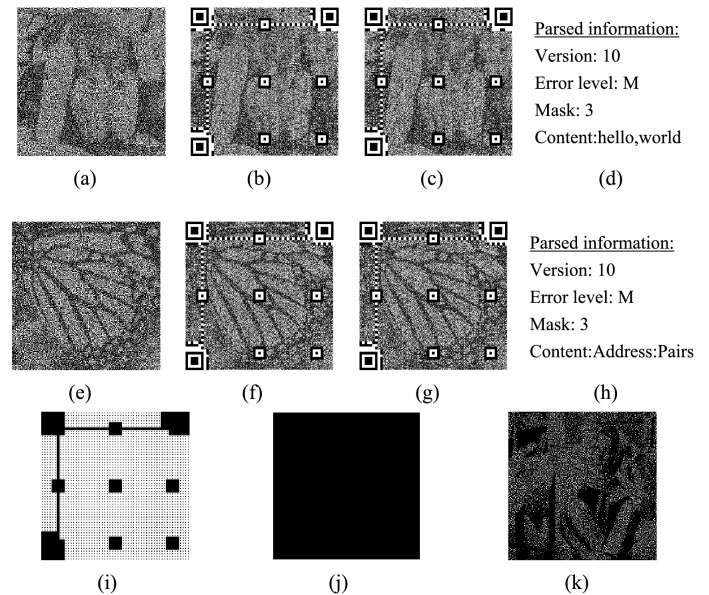
The experimental results of encryption, embedding, and recovery images. Halftoned cover images (**a**,**e**); cover images mixed with QR codes (**b**,**f**). Cover images embedded with overlayed pixels (**c**,**g**); parsed information with the zxing^16^ tool (**d**,**h**); mask image for QR codes (**i**); (**j**) is the difference between (**f**,**g**); recovery image (**k**).

Figure 3 shows the encrypted cover images and images mixed with meaningful shares and QR codes. Figure 3a–h is cover images mixed with QR codes and images embedded with overlayed pixels, and parsed information, respectively. Because QR codes are superimposed on the share shares, Fig. 3b(f) becomes darker compared with Fig. 3a(e). But Fig. 3c is visually unchanged. When the images of shares and QR codes are mixed, the black position in Fig. 3i will be overlapped by the pixels in QR codes, while the white areas will remain as the pixel in share images. Figure 3j is the difference image between Fig. 3f and g. Because we embed the overlapped pixels into the least significant bit of the pixels in QR codes, there is no difference between them. Figure 3k is the recovery image, which is the same as Fig. 2d.

**Figure 4:**
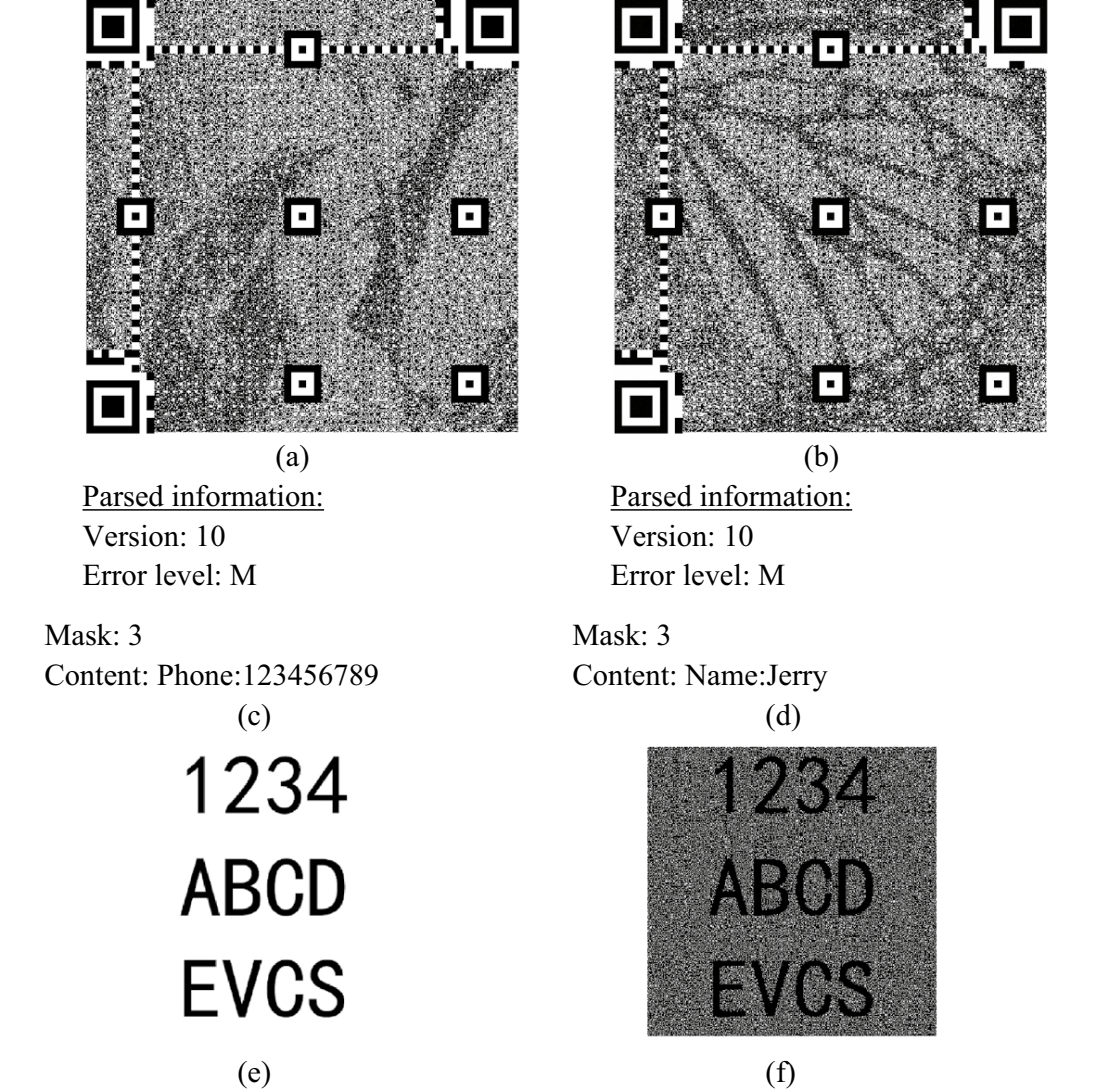
The experimental result of a plain text image. Cover images (**a**,**b**); parsed information by the zxing tool (**c**,**d**); secret image (**e**); recovery image (**f**).

We further evaluate the effectiveness of QEVCS on a plain text image. Figure 4a,b are QR code images that can be scanned and read normally. Parsed information is shown in Fig. 4c,d. Figure 4e,f are the secret and recovery image. As shown in Fig. 4, we can achieve good results on text images than normal images. This is because white pixels constitute the background, so its loss has little influence on image restoration. Benefiting from the property of perfect black of QEVCS, we can completely recover the black blocks that form secret characters.

We evaluate the metrics^17^ include SSIM (Structural Similarity Index Metric), PSNR (Peak Signal-to-Noise Ratio), and MSE (Mean Squared Error) for images recovery from the proposed scheme. The following table shows the performance of the proposed scheme for three secret images. The metric values for different QR codes versions are shown in Table 1. When one image is selected as the secret image, the other two images are used as cover images. The second column in Table 1 is the metrics values for the Barbara secret image, where Peppers and Butterfly are used for cover images. Since we have extended the QR codes size to fit the secret image size, the version of QR codes does not affect the image quality. All PSNR values are around 21, while the PSNR values are about 27 for the normal halftone images. To meet the security requirements, although our method reduces the image quality after binarization, the measurement results are roughly the same as the standard halftone technology, with a difference of about 6.

**Table 1:** The performance of the proposed scheme for three secret images.

version	Barbara			Peppers			Butterfly		
	PSNR	MSE	SSIM	PSNR	MSE	SSIM	PSNR	MSE	SSIM
8	21.12	502.44	6.23	21.01	515.32	6.23	21.23	489.87	6.22
10	20.12	632.53	6.24	20.20	620.98	6.24	20.32	604.06	6.23
14	21.34	477.62	6.24	21.49	461.40	6.25	21.39	472.15	6.24
16	21.19	494.40	6.28	21.59	450.90	6.28	21.23	489.87	6.26
30	21.89	420.80	6.30	22.10	400.94	6.29	22.31	382.01	6.28
35	22.40	374.18	6.29	22.41	373.32	6.31	22.52	363.98	6.32

Table 2 shows feature comparisons among our proposal and related works. Naor's revolutionary work has many shortcomings. Later work to try to solve some of these problems. Many methods combine QR codes and VCS. However, these methods use XOR operation, which makes it impossible for human eyes to restore secret images by simply superimposing images. Our method adopts the limited halftone method to present an expansion-free EVCS. At the same time, it keeps the meaning and printability (computation-free) of EVCS, which is not available in other methods.

**Table 2:** Feature comparisons among our proposal and previous schemes.

Scheme	Size-invariant	Meaningful	Transmission	Computation-free
Naor^6^	No	No	No	No
Pan^9^	Yes	No	Yes	No
Tan^11^	Yes	No	Yes	No
Cheng^13^	Yes	No	Yes	Yes
Zhang^18^	Yes	No	No	Yes
Zhang^19^	No	No	Yes	No
Our proposal	Yes	Yes	Yes	Yes

Many proposed schemes use the error correction function of QR codes to embed shared pixels. However, the fault tolerance rate of the highest level H of the error correction code is only 30%, that is, the damaged area of the two-dimensional code cannot exceed 30% of the whole image, which also limits the use of the whole two-dimensional code to transmit images. The error correction ability of the QR code can only reach the claimed error correction ratio in the case of continuous large-scale errors. For random noise errors, the error correction ability of the QR code is much lower than the claimed error correction ratio.

### Contrast analysis

The contrast of the image restored by our method is the same as that of the underlying EVCS. In the halftone processing of secret images, we use sub-pixels with 3/4 black pixels to represent white pixels, and sub-pixels with 4/4 black pixels to represent black pixels. The contrast of the image to be encrypted is 4/4–3/4 = 1/4. In the two cover images, we use 2/4 sub-pixels with black pixels to represent white pixels, 3/4 sub-pixels with black pixels, and the contrast of the processed cover images is 3/4–2/4 = 1/4.

### Security analysis

QEVCS can be divided into three steps. The first step is limited halftone processing, which is done independently by each image, so the information of the secret image will not be revealed. The second step is to encrypt the image with the underlying EVCS, which has been proved to be safe. The third step is pixel embedding, and this step is only related to the share images and QR codes, and will not reveal the information of the secret image. Therefore, QEVCS is secure.

## Conclusion

Visual cryptography perfectly combines the threshold characteristics of secret sharing with images, providing an effective solution to preserve the privacy of images. After printing shares on transparencies, HVS can recover the secret images without using any device. In this paper, we proposed a QR code-based expansion-free extended visual cryptography scheme (QEVCS). This scheme generates visually appealing QR codes for transmitting meaningful shares when keeping the printing friendliness of VCS. By the limited halftone and block encryption, QEVCS can reuse existing EVCS methods for constructing encryption matrices without pixel expansion. The experimental results show the effectiveness of QEVCS.

In the future, we will further improve the quality of restored images. To keep VCS friendly to HVS, our proposal sacrifices the contrast of images. Second, we will explore the combination of VCS with quantum computing^20^ since classical cryptography methods cannot resist quantum attacks. Last but not least, we are also working on the optimized pixel embedding method for overlaying shares into QR codes.
